# Survey on Actual Management of Osteoporosis with the Japanese Medical Data Vision Database in Elderly Patients Undergoing Spinal Fusion

**DOI:** 10.3390/jcm13102806

**Published:** 2024-05-10

**Authors:** Kenta Yamamoto, Shunichi Tanaka

**Affiliations:** Pharmaceutical R&D Business and Strategy Division, Musculoskeletal Pharmaceutical Brand Strategy, Asahi Kasei Pharma Corporation, 1-1-2 Yurakucho, Chiyoda-ku, Tokyo 100-0006, Japan; tanaka.scq@om.asahi-kasei.co.jp

**Keywords:** spinal fusion, osteoporosis, real-world data, peri-operative, assessment, DXA, treatment

## Abstract

**Background:** No actual data on spinal fusion and management of osteoporosis in Japan have been reported. The aim of the survey was to investigate pre- and post-operative management of osteoporosis, including testing and prescription, in elderly patients undergoing spinal fusion in Japan. **Methods:** Medical data on patients aged 65 years or older undergoing spinal fusion from April 2018 to March 2022 were extracted from the medical data vision (MDV) database containing health insurance claims data from Japanese acute care hospitals to investigate fusion area, pre- and post-operative osteoporosis tests (bone mineral density and osteoporosis markers), prescriptions of osteoporosis medications, and other information. **Results:** The analysis set consisted of 26,959 patients. Annual pre-operative BMD testing rates and osteoporosis markers testing rates were higher than the post-operative rates without significant annual changes. The post-operative prescription rate of osteoporosis medications throughout the target period was approximately two times higher than the preoperative rate. The drug with highest pre- and post-operative prescription rates was teriparatide (TPTD) followed by bisphosphonates, showing that the prescription rate of TPTD proportionally increased with the length of fusion area. **Conclusions:** It was suggested that patients aged 65 years or older undergoing spinal fusion might receive insufficient osteoporosis tests. Despite no trend in the testing rate with the length of fusion area, some tendency was observed in the selection of osteoporosis medications. In patients with osteoporosis undergoing spinal fusion, early examination, diagnosis, and therapeutic intervention may improve the prognoses, and solid testing and prescriptions are therefore expected.

## 1. Introduction

Although osteoporosis is highly likely to cause fragility fractures, treatment gaps still exist, which leads to deterioration in the activities of daily living, quality of life, and prognosis [1,2]. In Japan, which is a super-aged society, there were an estimated 15.9 million osteoporosis patients in 2015 [3]. Regarding in particular the prevalence of osteoporosis of L2–L4 or the femoral neck, the prevalence of osteoporosis in women aged ≥ 70 was 38.8%. Additionally, the prevalence of osteoporosis and incidence of bone fractures are higher than in other countries [4,5]. Thus, actions to reduce fragility fractures have been accelerated. For instance, expenses for continuous management to prevent secondary fractures after surgery for proximal femoral fractures were established by the revision of a regulation concerning medical fees [6].

In spinal fusion, osteoporosis may cause post-operative pseudarthrosis and pedicle screw loosening and increase the incidence of mechanical complications (e.g., cage subsidence) and proximal junctional kyphosis (PJK)/proximal junctional failure (PJF). Additionally, it has been considered an adverse prognostic factor, definitely leading to higher re-operation rates. Nevertheless, screening and treatment rates for osteoporosis during the peri-operative period of spinal operation remain low [7,8,9,10]. Some reports indicate that the screening implementation rate is only 19% [7]. Kadri et al. also reported a study that evaluated 124 patients aged 50 and over who were candidates for arthroplasty or thoracolumbar surgery. The results showed that osteoporosis (T-score of ≤−2.5) was present in 45% of women and 20% of men; only 3% of women and 10% of men had normal bone mineral density [11]. Recently, the concept of bone health optimization in orthopedics has been initiated for publication [11,12,13,14,15]. Particularly in spinal operations such as spinal fusion, guidelines for best practice in the peri-operative management of osteoporosis have been published [12,13,15]. A factor in common with those is that appropriate therapeutic interventions with pre-operative testing/assessment have been recommended in conditions accompanied by osteoporosis for elderly patients undergoing spinal fusion. In Japan, an aging society, patients undergoing a spinal operation are aging, and the complication rate of osteoporosis may therefore be increased in patients undergoing spinal fusion [16,17]. However, no actual data on spinal fusion and management of osteoporosis in Japan have been reported. Therefore, we used real-world data in Japan to examine the testing rates and interventions with osteoporosis medications in elderly patients undergoing spinal fusion.

## 2. Materials and Methods

### 2.1. Study Design and Data Source

This survey is a study using an existing anonymized database. Patient medical records and management data obtained from the MDV database (Medical Data Vision Co., Tokyo, Japan) were used. The MDV database contains administrative data on medical practices in inpatients and outpatients at hospitals participating in the Japanese Diagnosis–Procedure–Combination (DPC) system [18]. As of 1 April 2023, patient medical records and health insurance claims data from more than 460 hospitals reporting data on at least 40 million patients are included in the database. It is a large-scale database with accumulated data that have been anonymized after obtaining consent for secondary use of the data. The database contains the following data: patient demographics; claims for inpatient care; clinical diagnoses according to the tenth revision of the International Classification of Diseases; Japan’s unique standard disease codes; medical interventions; prescription data coded by the Anatomical Therapeutic Chemical classification of the World Health Organization; and patient claims data, including records on interventions with Japan’s unique standardized procedure codes.

### 2.2. Study Population

In the study, hospitals with data covering the entire period from 1 April 2017 to 31 March 2023, were targeted for data extraction. In those hospitals, data on men and women aged 65 years or older who underwent spinal fusion from 1 April 2018 to 31 March 2022 were included in the study.

Patients undergoing spinal fusion to be included in the analysis were defined as those who had the procedure code K142 (spinal fusion, laminectomy, and laminoplasty among the 21 procedures included in K142) and applied to any of four health insurance claim codes (150282510: anterior interbody fusion, 150282610: posterior or posterolateral interbody fusion, 150314610: posterior interbody fusion, and 150314710: simultaneous anterior/posterior interbody fusion). Details of operations included in the study are listed in Supplementary Table S1. Spinal fusion areas were classified into four categories: one level, two levels, three levels, and four or more levels.

Data on patients whose date of first targeted intervention was within the aggregation period from 1 April 2018 to 31 March 2022 were obtained, and the first implementation date was regarded as the index date. In patients who underwent an operation multiple times during the period, data on the date of the first operation conducted were obtained. When a target intervention was provided within 30 days, including the date of the first operation in those patients, the date of the latest intervention provided was regarded as the index date. When the date of the latest intervention provided within 30 days, including the first date, was due beyond the aggregation period, the first date was regarded as the index date. Moreover, the data were aggregated in the fiscal year ending in March.

### 2.3. Investigation Item

Data on conditions of surgical approaches, directions of fixation, and fusion areas (the number of intervertebral levels) for spinal fusion were aggregated. Furthermore, a bone mineral density (BMD) test and testing of osteoporosis markers (blood procollagen type 1 amino-terminal propeptide [P1NP], blood bone alkaline phosphatase [BAP], blood tartrate-resistant acid phosphatase 5b [TRACP-5b], urine N-terminal telopeptide [NTX], urine/blood beta type I collagen carboxy-terminal peptide, and blood 25-hydroxyvitamin D [25-OHD]) were investigated. Regarding prescriptions of osteoporosis medications, teriparatide (TPTD), romosozumab (ROMO), denosumab (Dmab), bisphosphonates (BPs), selective estrogen receptor modulator (SERM), and eldecalcitol (ELD) monotherapy were examined. Patients to whom ELD was prescribed with another osteoporosis medication were included in a group prescribed with drugs other than ELD. The efficacy of eldecalcitol monotherapy has been found to be higher than that of other forms of vitamin D monotherapy [19]. Thus, other active vitamin D preparations are mainly used in combination with other drugs, so they were not counted as monotherapy. A patient in whom an osteoporosis medication was switched during the period was counted as one patient both before and after switching the drug. The statuses of implementation of nutritional management, musculoskeletal rehabilitation, and psychotherapy were also investigated. Regarding psychotherapy, codes of interventions for mental health were extracted. Extracted data on codes of the tests, drugs, and interventions are listed in Supplementary Tables S2, S3, and S4, respectively.

The extraction period of data on the dates of BMD testing, testing of osteoporosis markers, and prescriptions for osteoporosis medications was specified to be within 360 days before and after the date of the implementation of spinal fusion. As for the BMD test, testing of osteoporosis markers, nutritional management, musculoskeletal rehabilitation, and psychotherapy, a patient who underwent the test or intervention multiple times was counted once. Additionally, for osteoporosis medications, the number of patients prescribed osteoporosis drugs was counted in two intervals: immediately after surgery (days 1–180) and after a certain period had elapsed (days 181–360).

### 2.4. Statistical Analysis

In the study, only aggregation was performed for extracted data, and statistical analyses were not conducted.

### 2.5. Ethical Approval

This study did not require institutional review board approval or patient consent. This is because the data that we used in this study consisted solely of hospital data for which permission for secondary usage was obtained, and MDV collected the data after they had been anonymized by the hospital in advance.

## 3. Results

Data from 333 hospitals reporting a total of 26,959 patients were extracted. The numbers of patients were 6330 patients in fiscal year (FY) 2018, 6675 in FY 2019, 6797 in FY 2020, and 7157 in FY 2021. The total numbers of patients who underwent spinal fusion by area fixed from FY 2018 to 2021 were 13,520 at one level, 5833 at two level, 2316 at three level, and 5290 at four or more levels (Table 1).

**Figure 1:**
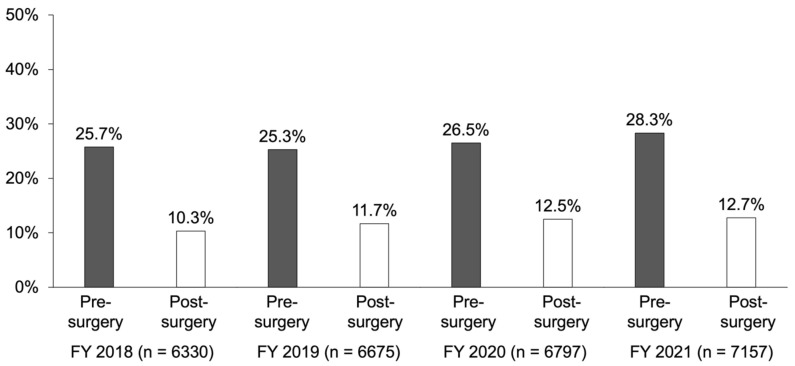
Annual changes in implementation rates of bone mineral density testing before and after surgery.

In patients undergoing spinal fusion, the annual pre-operative BMD testing rate was 25.7%, followed in order by 25.3%, 26.5%, and 28.3% from FY 2018 to 2021 (Figure 1). On the other hand, the post-operative BMD testing rate during the same period was 10.3%, followed in order by 11.7%, 12.5%, and 12.7%; the post-operative testing rate was less than half the pre-operative rate. The patients who underwent the test before and after surgery were duplicated, and the majority of patients undergoing the test after surgery also received the test before surgery.

**Figure 2:**
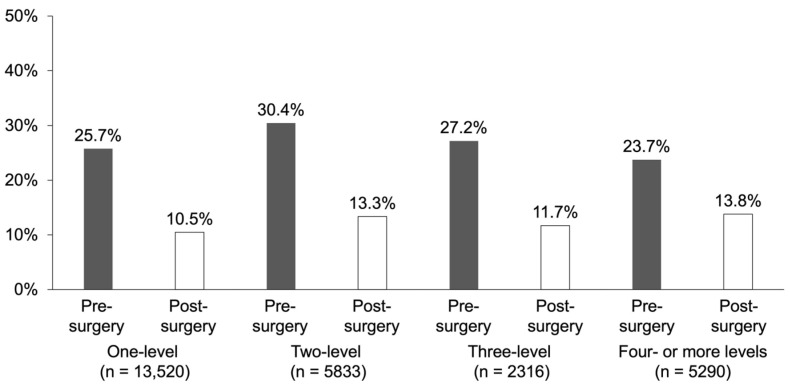
Implementation rates of bone mineral density testing by fusion area before and after surgery.

Based on the data by fusion area, patients undergoing two-level fusion surgery showed a higher pre-operative BMD testing rate. Moreover, a certain ratio of patients underwent the test after surgery, irrespective of the fusion area (Figure 2).

**Figure 3:**
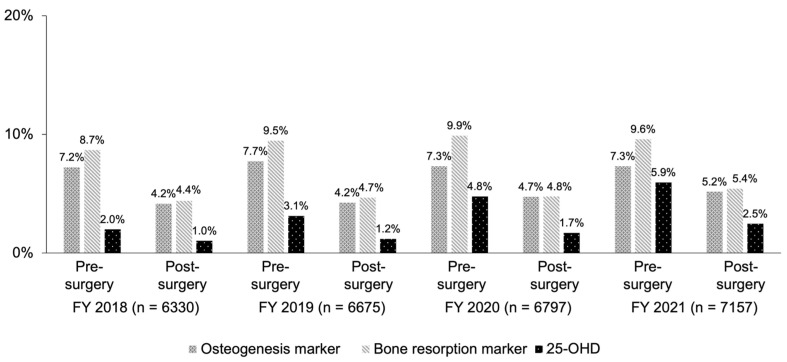
Annual changes in implementation rates of osteoporosis marker testing.

Annual changes in the status of implementation of bone metabolic marker measurements in patients with osteoporosis are indicated in Figure 3. The testing rates of osteogenesis markers remained unchanged from FY 2018 to 2021. The testing rates of 25-OHD, being lower than the rates of other markers, were continuously increased.

**Figure 4:**
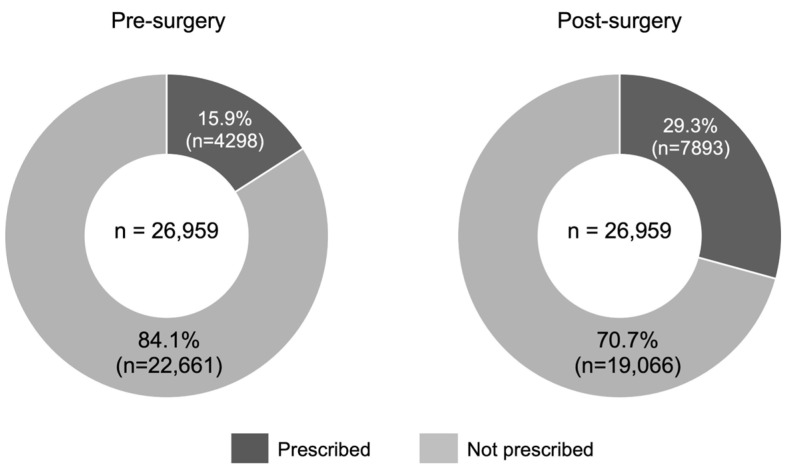
Ratios of patients prescribed a therapeutic agent for osteoporosis before and after surgery. Patients who were prescribed a therapeutic agent for osteoporosis at least once during the target period within 360 days before and after surgery were specified as those prescribed with the applicable drug (unique specification).

**Figure 5:**
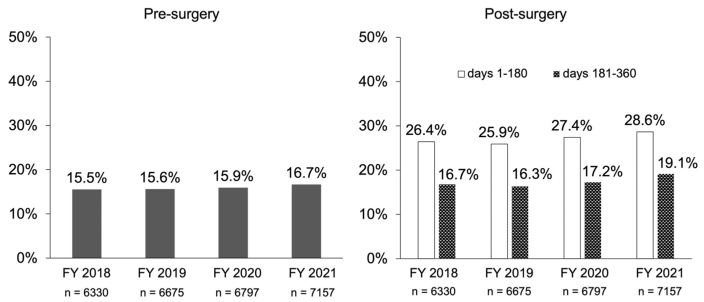
Annual changes in prescription rates of therapeutic agents for osteoporosis before and after surgery. Patients who were prescribed a therapeutic agent for osteoporosis at least once during the target period within 360 days before and after surgery were specified as those prescribed with the applicable drug (unique specification). In addition, post-operative days are divided into days 1–180 and 181–360.

Data on the presence or absence of prescriptions for osteoporosis medications are shown in Figure 4. The numbers of patients to whom one of monotherapy with TPTD, ROMO, Dmab, BPs, SERM, or ELD was prescribed at least once were 4298 out of 26,959 patients in total (15.9%) before surgery and 7893 (29.3%) after surgery; the number of those who were prescribed osteoporosis medication during days 1–180 post-surgery was about two times higher than the number before surgery. However, it returned to pre-surgery levels during days 181–360 post-surgery. The trend in annual prescription rates remained both before and after surgery from 2018 to 2021 (Figure 5).

**Figure 6:**
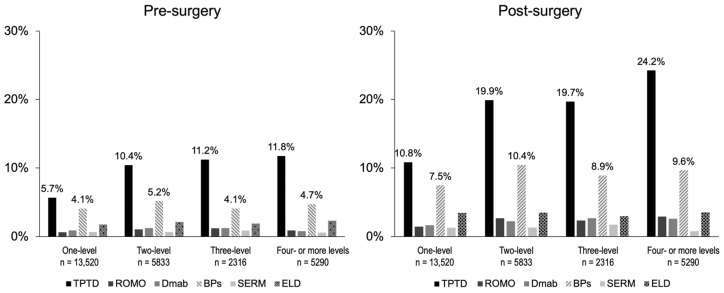
Patients who were prescribed a therapeutic agent for osteoporosis at least once during the target period within 360 days before and after surgery were regarded as those prescribed with the applicable drug.

The prescription rates of individual drugs by spinal fusion area throughout the target period are indicated in Figure 6. The drug with the highest prescription rate both before and after surgery was TPTD, followed by BPs. The prescription rate of TPTD increased with the increase in the number of intervertebral levels.

A patient in whom a drug was switched was counted twice as a patient prescribed with the drug both before and after switching the drug.

Patients to whom ELD was prescribed with another drug were counted as those prescribed with a drug other than ELD (TPTD, ROMO, Dmab, BPs, or SERM).

Statuses of implementation of nutritional guidance, exercise therapy, and psychotherapy before and after spinal fusion are indicated in Supplementary Table S5.

## 4. Discussion

This study suggests that the tests and treatments for osteoporosis are inadequate.

The BMD test is recommended in women aged 65 years or older and men aged 70 years or older [20,21,22,23,24]. The patients in our study were those aged 65 years or older undergoing spinal fusion, and the number of patients who did not receive the test was large. More BMD testing and diagnosis of osteoporosis may be required for appropriate treatment.

### 4.1. The Low Implementation rate of BMD Testing during the Peri-Operative Period of Spinal Fusion Surgery and Its Factors

It was reported that about 80% of surgeons experienced complications due to osteoporosis in spinal fusion; however, the ratio of surgeons who answered that they had conducted screening for osteoporosis was only 19% [7]. The BMD testing rate in the present study was slightly higher than the above result but was still low. Based on a questionnaire in a study of Japanese women without osteoporosis, 49% of the individuals had no history of BMD tests, and the proportion of those who answered that they felt at risk for osteoporosis in the future was only 15% [25]. In Japan, the government’s medical checkup service for health enhancement is currently being promoted to detect individuals with low bone mass and prevent osteoporosis in an earlier phase [26]. However, the ratio of those undergoing these medical checkups in 2021 was only 5.3% [27]. Lack of subjective symptoms of osteoporosis without fracture or pain may be associated with the low rates of medical examination and BMD testing.

The reason why the pre-operative BMD testing rate was at least two times higher than the post-operative rate was due to implant insertion during spinal fusion; this is because the test for lumbar spine BMD became unavailable after surgery. Moreover, considering that an operation is planned after understanding bone fragility by measuring BMD levels in advance of surgery, the pre-operative testing rate may be higher than the post-operative rate.

A systematic review and meta-analysis for the prevalence of osteoporosis in patients older than 50 years showed that a larger ratio of patients (78.7%) had osteoporosis/osteopenia among those undergoing a spinal operation [28]. Mo et al. reported that the underlying disease in most patients undergoing a spinal operation was spinal stenosis or lumbar spondylolisthesis, with complication rates of osteoporosis in patients aged 50 years or older being 54.2% in women and 39.5% in men [29]. Additionally, the report showed that rates of lumbar spondylolisthesis were 61.8% in women and 34.7% in men, where the rates increased with increasing age. In our study, the prescription rates of osteoporosis medications were 15.9% before surgery and 29.3% after surgery. Additionally, complication rates of osteoporosis were lower when compared to previous reports. However, such lower rates may be due to an absence of tests performed and not all osteoporosis patients being diagnosed. In studies by Fan et al. and Mo et al., the BMD test was performed in all patients. In our study, only one in four patients underwent the BMD test before and after surgery, indicating low implementation rates. In addition, in the elderly population (not limited to patients undergoing spinal fusion surgery), the prevalence of osteoporosis is extremely high in women as compared to men. However, according to the latest report from the Japanese Orthopedic Association National Registry (JOANR) for FY 2022, the number of patients undergoing spinal fusion surgery (posterior spinal interbody fusion) was 13,833 for males and 14,357 for females, with the sex ratio being nearly equal [30]. In this study, we were unable to extract sex data. If it is assumed that the sex ratio in this study is similar to that population, one possible factor influencing the low testing rates observed in this study could be the lower proportion of females as compared to the general population. However, since the study population originally consisted entirely of patients aged 65 and older, even higher testing rates are desirable. The best practice guideline for peri-operative management of osteoporosis recommends DXA testing for all patients over 65 years of age, regardless of sex [13]. There should be more potential osteoporosis patients, and they may be found by expanding BMD screening.

### 4.2. The Relationship between Screening Tests and History of Osteoporosis and Post-Operative Progression following Spinal Fusion Surgery

Gupta et al. and Mugge et al. reported that the incidence of implant-related complications and rate of re-operations were increased by osteoporosis as a complication after correction and fixation in adult spinal deformities [31,32]. Khalid et al. and Wolfert et al. reported that osteoporosis may be an adverse prognosis factor for post-operative implant-related complications and other conditions even after short fusion [33,34]. A report on spinal fusion and osteoporosis using big data in Japan by Nishida et al. showed a higher rate of re-operation and larger medical expenses in patients with osteoporosis as compared to those without the condition [35]. Raising the testing rate in patients undergoing spinal fusion with a potentially higher prevalence of osteoporosis may suppress the onset of post-operative complications. Additionally, it may improve the prognosis by identifying potential patients with osteoporosis and corresponding appropriate treatments before surgery. The number of tests in patients with osteoporosis may be insufficient in our study, and increasing them is desirable.

As for the relationship between the spinal fusion area and the BMD testing rate, the number of tests were expected to increase with the length of the fusion area. However, no significant difference in the testing rate due to the fusion area length was observed. One of the presumed reasons for this result was that the re-operation rate was very high in long spinal fusion [35], and osteoporosis may be diagnosed based on history of spinal fracture even without the BMD test.

In this study, the pre-operative testing rate of bone turnover markers was higher than the post-operative rate, as with the BMD test. Spinal fusion has been reported to change the levels of bone turnover markers [36]. Therefore, the test result not exactly reflecting the efficacy of a given osteoporosis medication might contribute to the lower testing rate after surgery. In the meantime, when the serum P1NP level is lower before surgery, a higher serum TRACP-5b level has been reported to enhance nonunion of bone after spinal fusion [37]. For this reason, pre-operative bone turnover marker tests with follow-up during treatment of osteoporosis may play an important role in the prognosis and outcome of patients undergoing spinal fusion with a higher prevalence of osteoporosis. For users of osteoporosis drugs, it is necessary to reassess markers after surgery to determine their effectiveness. In practice, bone turnover markers other than 25-OHD and serum calcium concentration are not entirely necessary at the stage of diagnosing osteoporosis, so not all patients undergoing spinal fusion surgery are subject to testing. However, examining the preoperative testing rate would provide one of the factors for estimating the scale of patients receiving osteoporosis treatment.

As for 25-OHD, Ravindra et al. reported that 30.0% of patients undergoing selective spinal fusion had a vitamin D deficiency [38]. Stoker et al. showed that 27.0% of patients undergoing spinal fusion had a vitamin D deficiency [39]. Moreover, Kim et al. found that the functional outcome after spinal fusion was inversely related to baseline 25-OHD at the time of spinal fusion [40]. Therefore, measurement of 25-OHD is significant in patients undergoing spinal fusion.

From the findings above, measurement of bone turnover markers in patients with osteoporosis may allow them to serve as predictors of prognoses in spinal fusion. Thus, preoperatively performing testing in most patients who are definitively diagnosed with osteoporosis may lead to appropriate therapeutic intervention before surgery and improvement of the prognosis.

### 4.3. Trends and Factors in the Selection of Osteoporosis Treatment Drugs in Patients Undergoing Spinal Fusion Surgery

Aggregation of data on osteoporosis medications by fusion area showed that the post-operative prescription rates of most of drugs were approximately two times higher than pre-operative rates, and the most commonly prescribed drug was TPTD. The prescription rate of TPTD was higher in patients who underwent multi-vertebral surgery. It may be associated with enhancement of bone union by TPTD administration in the peri-operative period of spinal fusion. It results in decreased rates of the onset of mechanical complications such as pedicle screw loosening and PJK/PJF, as well as improvement of post-operative quality of life reported in many articles [41,42,43,44,45,46]. Despite its high cost, TPTD is prescribed the most in Japan for patients undergoing spinal fusion surgery during the peri-operative period, and this can be attributed to two main reasons. Firstly, there is a substantial body of evidence in Japan demonstrating the beneficial effects of TPTD in patients with osteoporosis who undergo spinal fusion surgery [41,42,43,44,45]. Additionally, neoadjuvant TPTD has been reported to be a cost-effective strategy for reducing post-operative complications in patients with osteopenia undergoing adjacent segment degeneration surgery [47]. Secondly, Japan has a comprehensive healthcare insurance system, the National Health Insurance system. It reduces the amount that patients pay and makes it easier to use such medications. Therefore, at present, this trend in drug selection may be unique to Japan.

Furthermore, while the prescription rate of osteoporosis medications during days 1–180 post-surgery increased compared to pre-surgery, it returned to pre-surgery levels during days 181–360 post-surgery. It has been reported that the rate of continuation of osteoporosis medications decreases to 50–60% over the course of one year [48]; this is a longstanding issue requiring resolution. Discussions on the necessity of peri-operative medications and the promotion of treatment continuation will be conducted more actively.

In this study, we calculated the proportion of tests and prescriptions using all patients who underwent spinal fusion surgery as the denominator, without limiting the data analysis to patients diagnosed with osteoporosis. This revealed a low rate of BMD testing, suggesting that not all patients with osteoporosis have been definitively diagnosed. Basically, if osteoporosis is definitively diagnosed, most patients will be eligible for drug treatment. Therefore, patients prescribed osteoporosis treatment drugs are almost equivalent to those definitively diagnosed with osteoporosis. Taking this into account, the low prescription rate of osteoporosis drugs in patients undergoing spinal fusion surgery indicates a potential failure to identify an adequate number of osteoporosis patients. If BMD testing was conducted in all patients aged 65 and older, we would more likely know the accurate proportion of osteoporosis patients among patients undergoing spinal fusion surgery and the corresponding prescription patterns within that group. Therefore, even in cases where elderly patients undergoing spinal fusion surgery have never undergone BMD testing, it is desirable that BMD testing is conducted at least once before surgery. The global guidelines for spinal surgery and osteoporosis recommend that all patients over 65 years of age undergo BMD testing [12,13], but there is still a large gap between actual clinical practice in Japan and these recommendations. Identifying potential osteoporosis patients would likely lead to the initiation of appropriate treatment early on and improve post-operative outcomes.

In Japan, which is super-aged society, identification of patients with osteoporosis and therapeutic interventions are urgently required. With the concomitant increase in medical care costs, it is necessary to prevent fractures and prevent patients becoming bedridden due to osteoporosis.

This study has some limitations. Most of the medical institutions registered in the MDV database are large-scale hospitals specializing in acute-phase treatments. That is, the patients and conditions indicated for spinal fusion may be different from those at small- and medium-scale general hospitals. Furthermore, only data from hospitals participating in the DPC system were included in the database. In other words, medical record data on a patient who had usually visited a personal orthopedist and then been referred to a target hospital to undergo an operation could not be extracted in this study. Despite the presence of significant gender differences in osteoporosis, no gender data were extracted, and no effects of gender on the tests and prescriptions were examined. The study population consisted of patients who underwent spinal fusion surgery and not all patients diagnosed with osteoporosis. Therefore, we have to take into account that our results do not only concern the examination and prescription rates of osteoporosis patients; such points were included in the limitations of the study.

## 5. Conclusions

It was suggested that patients aged 65 years or older undergoing spinal fusion might not receive sufficient BMD testing for diagnosing osteoporosis and implementing follow-up. For osteoporosis patients undergoing spinal fusion, early examination, diagnosis, and therapeutic intervention may improve prognoses. Therefore, adequate testing and prescriptions are recommended. Specifically, for patients without a history of BMD testing in the year preceding surgery, it would be beneficial to include BMD testing in clinical pathways for the peri-operative period of spinal fusion surgery.

## Figures and Tables

**Table 1:** Annual changes in number of patients undergoing spinal fusion by fusion area.

	2018	2019	2020	2021	Total
Spinal fusion area (n)					
One level	3206	3331	3351	3632	13,520
Two levels	1471	1478	1414	1470	5833
Three levels	564	570	607	575	2316
Four or more levels	1089	1296	1425	1480	5290
Total	6330	6675	6797	7157	26,959

## Data Availability

The data that support the findings of this study are available from MDV Co., Ltd.; however, these were used under the license for the current study and are therefore not publicly available.

## Supplementary Materials

The following supporting information can be downloaded at: https://www.mdpi.com/article/10.3390/jcm13102806/s1, Supplementary Table S1: List of techniques for spinal fusion to be extracted, Supplementary Table S2: List of osteoporosis markers to be extracted, Supplementary Table S3: Osteoporosis medication, Supplementary Table S4: List of nutritional management, musculoskeletal rehabilitation, and psychotherapy to be extracted, Supplementary Table S5: Execution of nutritional management, musculoskeletal rehabilitation, and psychotherapy by fusion area before and after surgery.
