# Supplementary material for: Survey on Actual Management of Osteoporosis with the Japanese Medical Data Vision Database in Elderly Patients Undergoing Spinal Fusion
Source: J Clin Med. 2024 May 10;13(10):2806. doi: 10.3390/jcm13102806 (PMC11121966; doi:10.3390/jcm13102806)
Supplement: Supplementary file 1 [file jcm-13-02806-s001.zip › Supplementary Table.pdf]

Supplementary Table S1. List of techniques for spinal fusion to be extracted.

Classification code	Category name	Health insurance claim code	Name of health insurance claim code
K142	Spinal fusion, laminectomy, and laminoplasty (surgeries in regions between multiple disc spaces and vertebral arches are included)	150282510	Spinal fusion, laminectomy, and laminoplasty (anterior interbody fusion)
		150282610	Spinal fusion, laminectomy, and laminoplasty (posterior or posterolateral fusion)
		150314610	Spinal fusion, laminectomy, and laminoplasty (posterior interbody fusion)
		150314710	Spinal fusion, laminectomy, and laminoplasty (simultaneous anterior/posterior interbody fusion)

Supplementary Table S2. List of osteoporosis markers to be extracted.

Classification code	Category name	Health insurance claim code	Name of health insurance claim code
D008	Endocrinology test	160168650	BAP
		160201750	P1NP
		160164250	NTX
		160184450	TRACP-5b
		160174850	Beta-CTX (urine)
		160181050	Beta-CTX
D007	Blood chemistry test	160215350	25-hydroxyvitamin
		160209050	25-hydroxyvitamin D

Supplementary Table S3. Osteoporosis medication.

ATC (EphMRA)	Non-proprietary name	Dosage form
A11C2	Eldecalcitol	Oral
M05B3	Alendronate sodium hydrate	Oral, injection
	Ibandronate sodium hydrate	Oral, injection
	Zoledronic acid hydrate	Injection
	Minodronic acid hydrate	Oral
	Sodium risedronate hydrate	Oral
G03J0	Bazedoxifene acetate	Oral
	Raloxifene hydrochloride	Oral
H04E0	Abaloparatide acetate	Injection
	Teriparatide acetate	Injection
	Teriparatide (recombinant)	Injection
M05B9	Romosozumab (recombinant)	Injection
	Denosumab (recombinant)	Injection

Supplementary Table S4. List of nutritional management, musculoskeletal rehabilitation, and psychotherapy to be extracted.

	Classification code	Category name	Health insurance claim code	Name of health insurance claim code
Nutritional management	A104	Basic daily fee for hospitalization at special functioning hospitals	190240210	Fee to be added for nutritional management system during hospital admission (basic fee for hospitalization at special functioning hospitals)
	A108	Basic daily fee for hospitalization at clinics with beds	190170770	Fee to be added for nutritional management (basic fee for hospitalization at clinics with beds)
	A109	Basic daily fee for long-term care beds at clinics with beds	190171770	Fee to be added for nutritional management (basic fee for long-term care beds at clinics with beds)
	A233	Daily fee to be added for nutritional management	190120410	Fee to be added for nutritional management
	A233-2	Weekly fee to be added for the nutritional support team	190136810	Fee to be added for the nutritional support team
			190147110	Fee to be added for the nutritional support team (specific area)
			190191770	Fee to be added for cooperation with dentist
	K000	Wound treatment	150425190	Fee to be added for peri-operative nutritional management
	A100	Basic daily fee for hospitalization in the general hospital ward	190182590	Specified fee to be subtracted for the nutritional management system (basic fee for hospitalization)
			190182690	Specified fee to be subtracted for the nutritional management system (specific fee for hospitalization)
Musculoskeletal rehabilitation	H002	Fee for musculoskeletal rehabilitation	180023610	Occupational therapy (1) (individualized therapy)

	180023710	Occupational therapy (1) (group therapy)
	180032710	Fee for musculoskeletal rehabilitation (1)
	180044130	Fee for musculoskeletal rehabilitation (2) (nursing care required) Not conformed to the standard
	180044230	Fee for musculoskeletal rehabilitation (3) (nursing care required) Not conformed to the standard
	180052730	Fee for musculoskeletal rehabilitation (1)*
	180052830	Fee for musculoskeletal rehabilitation (2)*
	180052930	Fee for musculoskeletal rehabilitation (3)*
	180053030	Fee for musculoskeletal rehabilitation (1) (nursing care required / inpatient)*
	180053130	Fee for musculoskeletal rehabilitation (1) (nursing care required / outpatient)*
	180053230	Fee for musculoskeletal rehabilitation (2) (nursing care required / inpatient)*
	180053330	Fee for musculoskeletal rehabilitation (2) (nursing care required / outpatient)*
	180053430	Fee for musculoskeletal rehabilitation (3) (nursing care required / inpatient)*
	180053530	Fee for musculoskeletal rehabilitation (3) (nursing care required / outpatient)*
	180053630	Fee for musculoskeletal rehabilitation (1) (nursing care required) Not conformed to the standard*
	180053730	Fee for musculoskeletal rehabilitation (2) (nursing care

		required) Not conformed to the standard*
	180023810	Occupational therapy (2) (individualized therapy)
	180023910	Occupational therapy (2) (group therapy)
	180027810	Fee for musculoskeletal rehabilitation (2)
	180053830	Fee for musculoskeletal rehabilitation (3) (nursing care required) Not conformed to the standard*
	180024070	Fee to be added for early rehabilitation during occupational therapy (A)
	180024170	Fee to be added for early rehabilitation during occupational therapy (B)
	180024270	Fee to be added for early rehabilitation during occupational therapy (C)
	180024370	Fee to be added for early rehabilitation during occupational therapy (A) (patients aged less than 15 years)
	180024470	Fee to be added for early rehabilitation during occupational therapy (B) (patients aged less than 15 years)
	180024570	Fee to be added for early rehabilitation during occupational therapy (C) (patients aged less than 15 years)
	180024670	Fee to be added for early rehabilitation during occupational therapy (e.g., early ambulation and independence in ADL)
	180027910	Fee for musculoskeletal rehabilitation (3)
	180029510	Fee for musculoskeletal rehabilitation (1) (more than 120 days)
	180029610	Fee for musculoskeletal rehabilitation (2) (more than 120

				days)
			180034510	Fee for musculoskeletal rehabilitation (1) (nursing care required / outpatient)
			180045810	Fee for musculoskeletal rehabilitation (1) (nursing care required / inpatient)
			180034610	Fee for musculoskeletal rehabilitation (2) (nursing care required / outpatient)
			180045910	Fee for musculoskeletal rehabilitation (2) (nursing care required / inpatient)
			180034710	Fee for musculoskeletal rehabilitation (3) (nursing care required / outpatient)
			180046010	Fee for musculoskeletal rehabilitation (3) (nursing care required / inpatient)
			180043370	Fee to be subtracted for non-conformity to facility standards (expense for musculoskeletal rehabilitation; ninety hundredth)
			180044030	Fee for musculoskeletal rehabilitation (1) (nursing care required) Not conformed to the standard
Psychotherapy	I002	Psychotherapy per consultation on an outpatient basis / at home	180067230	Psychotherapy on an outpatient basis (at least 60 minutes on the first consultation day / fee reduction depending on the requirement; other than Item 1)
			180067530	Psychotherapy on an outpatient basis (less than 30 minutes / fee reduction depending on the requirement; other than Item 1)

		180067630	Psychotherapy on an outpatient basis (at least 30 minutes / fee reduction depending on the requirement; other than Item 1)
		180071530	Psychotherapy on an outpatient basis (at least 60 minutes on the first consultation day / fee reduction depending on the requirement; designated psychiatrist)
		180071630	Psychotherapy on an outpatient basis (at least 30 minutes / fee reduction depending on the requirement; designated psychiatrist)
		180071730	Psychotherapy on an outpatient basis (less than 30 minutes / fee reduction depending on the requirement; designated psychiatrist)
I002-2	Daily support / instruction fee for continuous treatment at the Department of Psychiatry on an outpatient basis	180031410	Support / instruction fee for continuous treatment at the Department of Psychiatry on an outpatient basis
		180031570	Fee to be added to support environmental improvement for medical treatment
		180035770	Fee to be subtracted in case of three or more drugs prescribed (antianxiety or hypnotic)
		180035870	Fee to be added for evaluation of adverse reactions by specific drugs (support / instruction fee for continuous treatment at the Department of Psychiatry on an outpatient basis)
		180050230	Support / instruction fee for continuous treatment at the

			Department of Psychiatry on an outpatient basis (fee reduction depending on the requirement; Note 2 is excluded)
I002-3	Support fee for continuous treatment at the Department of Psychiatry in emergency patients	180047610	Support fee for continuous treatment at the Department of Psychiatry in emergency patients (inpatients)
		180047710	Support fee for continuous treatment at the Department of Psychiatry in emergency patients (outpatients)

*Health insurance claim code when the fee was reduced due to not fulfilling a certain condition specified by the regulatory agency in patients undergoing musculoskeletal rehabilitation.

Supplementary Table S5. Numbers of Executions of Nutritional Management, Musculoskeletal Rehabilitation, and Psychotherapy by Fusion Area Before and After Surgery.

	2018		2019		2020		2021		2022	
Pre-/post-surgery	Pre-surgery	Post-surgery	Pre-surgery	Post-surgery	Pre-surgery	Post-surgery	Pre-surgery	Post-surgery	Pre-surgery	Post-surgery
Nutritional management										
Total	6,257	6,330	6,589	6,675	6,708	6,797	7,035	7,157	26,589	26,959
One-level	3,197	3,206	3,307	3,331	3,334	3,351	3,596	3,632	13,434	13,520
Two-level	1,463	1,471	1,467	1,478	1,400	1,414	1,458	1,470	5,788	5,833
Three-level	557	564	554	570	598	607	561	575	2,270	2,316
Four- or more levels	1,040	1,089	1,261	1,296	1,376	1,425	1,420	1,480	5,097	5,290
Musculoskeletal rehabilitation										
Total	3,041	5,304	3,251	5,638	3,572	5,692	3,645	6,034	13,509	22,668
One-level	1,558	2,702	1,579	2,850	1,761	2,860	1,842	3,101	6,740	11,513
Two-level	695	1,247	764	1,291	777	1,221	773	1,272	3,009	5,031
Three-level	263	481	270	463	305	476	276	469	1,114	1,889
Four- or more levels	525	874	638	1,034	729	1,135	754	1,192	2,646	4,235
Psychotherapy										
Total	125	276	139	328	132	323	144	530	540	1,457
One-level	43	87	48	109	48	123	51	224	190	543
Two-level	25	66	35	71	28	61	36	99	124	297
Three-level	19	28	10	33	9	29	13	51	51	141

Four- or more levels	38	95	46	115	47	110	44	156	175	476
